# Efficacy of Active Charcoal and Mannitol in Patients with Haff Disease Caused by the Consumption of Crayfish (*Procambarus clarkii*): A Retrospective Cohort Study

**DOI:** 10.1155/2020/2983589

**Published:** 2020-09-14

**Authors:** Jian Lv, Qun Zhou, Shuangle Wang, Fengqin Wei, Xiaozheng Zhang, Luyao Zhang, Haibin Ni

**Affiliations:** ^1^Emergency Department, Affiliated Hospital of Integrated Traditional Chinese and Western Medicine, Nanjing University of Chinese Medicine, 100 Shizi Road, Nanjing 210028, China; ^2^Department of Pathology, School of Medicine & Holistic Integrative Medicine, Nanjing University of Chinese Medicine, 138 Xianlin Road, Nanjing 210023, China

## Abstract

This study evaluates the clinical efficacy of activated charcoal combined with mannitol (ACM) for the treatment of Haff disease. This is a retrospective cohort study conducted at the Emergency Department of Affiliated Hospital of Integrated Traditional Chinese and Western Medicine. Consecutive patients who were hospitalized during a two-year time frame (from June 2016 to August 2017) and diagnosed with Haff disease were reviewed. Clinical symptoms, laboratory findings, pain/anxiety scores, and treatment-related adverse events were collected. Sixty-eight Haff disease patients after boiled crayfish consumption were enrolled in this study. Besides standard treatments for Haff disease, 22 patients had an oral administration of activated charcoal and mannitol within 12 hours of hospital admission (ACM group), while the other 46 patients did not receive such treatment (non-ACM group). Baseline characteristics including clinical symptoms, serum enzyme levels, and pain/anxiety scores were comparable between the two groups. Activated charcoal and mannitol treatment led to lower CK-MB and AST levels from 12 hours to 60 hours, lower ALT and LDH levels from 12 hours to 72 hours, and lower CK levels from 24 hours to 72 hours after hospitalization. Patients in the ACM group had significantly shortened duration of hospital stays (7.5 [6.0–8.0] days vs 8.0 [6.8–10.0] days, *p* = 0.032) and lower anxiety scores 24 hours after hospital admission (40.7 ± 4.9 vs 44.1 ± 6.3, *p* = 0.032) than in the non-ACM group. No patient experienced treatment-related adverse events. The overall prognosis of both groups is good. Among patients with Haff disease caused by boiled crayfish, activated charcoal combined with mannitol treatment resulted in shorter hospital stays, lower serum CK, CK-MB, AST, ALT, and LDH levels, and lower anxiety scores.

## 1. Introduction

Haff disease is a rare syndrome of unexplained myalgia and rhabdomyolysis in a person who ate certain types of cooked freshwater fish or crustacean in the 24 hours before onset of illness [1]. Currently, a yet unidentified heat-stable toxin is believed to be the cause. Haff disease was first described in 1924 in Russia following the consumption of freshwater eel (*Anguilla anguilla*), pike (*Esox* species), and, especially, burbot (*Lota lota*) [2]. After that, outbreaks of Haff disease were reported in Sweden, the former Soviet Union, the United States, and also the Brazilian Amazon [3].

Reports of Haff disease outbreaking in China are uncommon. Yuen and Chen first reported that six patients in Beijing presented Haff disease after consumption of crayfish (*P*. *clarkii*) in 2000 [4]. Afterwards, an outbreak of unexplained myalgia after consumption of freshwater pomfrets (*Colossoma brachypomum*) occurred on 27 October 2009, in a town of Lianzhou County, Guangdong Province, South China [5]. One hundred and fifty-nine subjects consumed freshwater pomfrets on 26 October 2009, of whom 50 fell ill. Although they showed symptoms of weakness, myalgia, nausea, and abdominal pain, all patients recovered without sequelae soon. However, the following toxin analyses of pomfret samples, water, and soil samples were negative. A Haff disease outbreak between July and August 2010, caused by crayfish involving 23 cases, was reported in Nanjing, Jiangsu Province, East China [6]. In consistent with the outbreak early in 2009, no possible toxins, drugs, and hazardous elements were found from crayfish samples. As eating boiled crayfish grew prevalent in Southeast China, Haff disease patients suddenly shot up in 2016, when 494 cases were reported from 15 different hospitals in Nanjing during 5 July to 29 August, 2016 [7]. This outbreak was associated with consumption of crayfish produced from the Yangtze River and its surrounding water system. Again, chemical analysis of crayfish, river water, and sediment did not identify known or novel toxins. Affiliated Hospital of Integrated Traditional Chinese and Western Medicine, located on northeast of Nanjing City, is an 830-bed tertiary medical facility. The emergency department of our hospital serves about 4000 patients every day. Since 2016, the number of patients diagnosed with Haff disease presenting in our emergency is surging. Studies that reported this outbreak focused on epidemiological investigation, and few studies tried to explore the treatment for these patients. It is generally believed that Haff disease is caused by a heat-stable toxin. Activated charcoal can absorb the toxins in the gastrointestinal tract and mannitol administered orally may favor fast colonic transit, thus accelerate the evacuation of toxins. In clinical practice, we tried activated charcoal and mannitol for treatment of these patients, and we suppose this treatment might be useful for alleviating disease severity.

In this retrospective cohort study, we assessed the efficacy of activated charcoal combined with mannitol in treating Haff disease. The hypothesis we hold is that this treatment is effective in decreasing serum creatine kinase (CK) and creatine kinase-muscle/brain (CK-MB) levels and relieving symptoms of Haff disease patients.

## 2. Methods

This study was approved by the Ethical Committee of Affiliated Hospital of Integrated Traditional Chinese and Western Medicine. This study did not interfere with treatments for these patients, and we did not obtain any additional samples or data from the patients. Hence, written informed consent was not required.

### 2.1. Patients and Selection Criteria

From the Electronic Medical Record System at our hospital, we retrospectively reviewed 71 consecutive adult patients who ate boiled freshwater crayfish within 24 hours before onset of Haff disease admitted to our emergency department from June 2016 to August 2017. The diagnostic criteria for Haff disease were as follows: (1) patients presented with rhabdomyolysis-like symptoms, such as (sub)acute-onset myalgia, transient muscle weakness, or hematuria and (2) laboratory results revealed a fivefold or greater elevation in CK levels with an MB fraction < 5% [8]. The exclusion criteria included patients who had other diseases with elevated CK levels, such as acute coronary syndrome and who had a recent history of trauma or intense exercise and incomplete clinical data. Three patients were excluded: two patients had a recent history of trauma and one patient had incomplete clinical data. Ultimately, sixty-eight patients were enrolled in this study.

### 2.2. Treatment and Pain/Anxiety Assessment

All patients received early and adequate volume replacement with NaCl 0.9% intravenously administered 2000 mL/d and warm water orally taken 1000–2000 mL/d to maintain a urine output of 100–150 mL/h. Sodium bicarbonate 5% was given 125–250 mL/d for alkalization of the urine. Patients also received treatment for stomach and liver protection [9]. Twenty-two patients took additional activated charcoal (30 g, mixed in 200 mL water) and mannitol (20%, 250 mL) orally. These patients received activated charcoal and mannitol treatments once 12 hours within hospital admission.

Visual Analogue Scale (VAS) was used to assess the pain. The patients were instructed to score the maximal intensity of pain experienced on hospitalization and 24 hours after admission over a scale of 0 to 10 [10]. Intravenous remifentanil was delivered to alleviate pain if the pain score exceeded 6. The level of anxiety was assessed by the Zung Self-Rating Anxiety Scale (SAS) at admission and 24 hours after hospitalization, respectively [11]. Each scale contains 20 items based on a 0–4 scale, with a higher score indicating a higher level of anxiety. SAS scores below 50 were normal, while 50 to 59 stand for mild anxiety, 60 to 69 moderate, and 70 to 80 severe.

The treatment-related adverse events included pulmonary aspiration, abdominal distention, severe diarrhea, incomplete intestinal obstruction, constipation, and electrolyte imbalances. Adverse events were calculated throughout hospital stays.

### 2.3. Data Collection

Clinical data including the patients' demographic characteristics and the initial presenting symptoms were collected from the Electronic Medical Record System at our hospital. Blood from veins were collected from patients on hospital admission and every day at 6: 00 am during hospitalization. Laboratory data including serum CK, CK-MB, aspartate transaminase (AST), alanine transaminase (ALT), and lactic dehydrogenase (LDH) levels were detected by the clinical laboratory of our hospital.

### 2.4. Statistics

Data were statistically analyzed with SPSS (version 23.0; IBM, USA). Categorical variables were presented as counts with percentages. Continuous variables with a normal distribution were presented as means ± standard deviations (SD) and nonnormal distribution as medians and interquartile ranges (IQR). To compare serum enzymes over 72 hours, the repeated measures ANOVA was used. To compare the groups, a *t*-test was conducted against the continuous data and the categorical data were analyzed with a *X*^2^. A two-tailed *p* value of less than 0.05 was considered statistically significant.

## 3. Results

### 3.1. Epidemiology Characteristics

The study included 68 patients with Haff disease admitted in our department stretching from June 2016 to August 2017, consisting of 22 patients treated with activated charcoal combined with mannitol (ACM group) and 46 patients as controlled group (non-ACM group) (Table 1). In the ACM group, there were 10 females and 12 males with an average age of 34.4. In the non-ACM group, there were 26 females and 20 males with an average age of 33.6. No significant difference was found between groups in age (*p*=0.731) and gender distribution (*p*=0.392). The duration of crayfish consuming prior to symptoms onset was 3.4 ± 1.7 hours in the ACM group and 3.9 ± 2.0 hours in the non-ACM group (*p*=0.296). The time interval from boiled crayfish consumption to the ACM treatment start is 5.2 [4.5–7.5] hours. The ACM group showed shorter in-hospital stays than the non-ACM group (7.5 [6.0–8.0] days and 8.0 [6.8–10.0] days, *p*=0.032). All subjects fully recovered after treatment, and no organ failure occurred.

### 3.2. Clinical Characteristics on Admission

Myalgia and weakness were two most common initial syndromes, presenting in every patient (Table 1). Other syndromes included shortness of breath (13 patients, 19%), nausea or vomiting (9 patients, 13%), abdominal pain (7 patients, 10%), and hematuria (7 patients, 10%). No statistical difference was observed in clinical syndromes between the two groups.

Blood samples were collected at the time of first presentation in every subject. Analysis indicated that levels of CK, CK-MB, AST, ALT, and LDH were all elevated (Table 2). Serum creatinine levels in all patients were in a normal range, while 25 patients showed slight elevation of blood urea nitrogen (BUN) levels. Also, there was no significant statistical difference in serum initial enzyme levels between two groups.

### 3.3. Changes in Serum Enzymes over 72 Hours after Admission

As shown in Table 2, the serum levels of CK peaked 12 hours (10354.1 ± 3623.7 U/L) after hospital admission and gradually returned to 156.5 ± 47.3 U/L within 72 hours in the ACM group. In the non-ACM group, the serum CK levels reached a peak value of 9509.4 ± 4149.1 U/L at 12 hours and decreased to 5443.3 ± 2224.6 U/L at 24 hours. However, CK levels increased again to 5465.0 ± 1536.0 U/L at 36 hours and then declined to 850.3 ± 263.1 U/L at 72 hours in the non-ACM group. The ACM group showed significantly greater reduction than the controls on CK levels at 24 hours, and this reduction was maintained afterwards throughout the study. Serum CK-MB, AST, ALT, and LDH values elevated and reached peak values on hospitalization and then reduced quickly within 72 hours in all subjects. All these levels were consistently higher in the non-ACM group patients than the ACM group patients after admission.

### 3.4. Assessment of Pain and Anxiety

As shown in Table 3, the baseline pain and anxiety scores were comparable between the two groups and both scores decreased significantly after 24 hours of treatment (*p* < 0.001). Patients that received ACM treatment had less anxiety scores at 24 hours (40.7 ± 4.9 vs 44.1 ± 6.3, *p*=0.032). However, pain scores at 24 hours did not show statistical differences between the two groups (1.1 ± 0.9 vs 1.4 ± 1.1, *p*=0.296). In addition, no subject in the ACM group experienced treatment-related adverse events.

## 4. Discussion

Crayfish are freshwater benthic animals, and more than 540 species of crayfish have been discovered in the world. In China, crayfish show their vigorous growth and reproduction in the areas from the middle and lower reaches of the Yangtze River for its favorable ecological conditions [12]. Crayfish soon gain its popularity among people living in Nanjing, to be specific, from June to September every year, and they consume approximately 60 to 80 tons of crayfish every day [6, 13]. Unfortunately, as poisonous or toxic pollutants in the river increase sharply these years, and the food chain of crayfish has changed dramatically [14]. The etiology of rhabdomyolysis in Haff disease is still not clear. Chemical analysis of crayfish, river water, and sediment did not find known or novel toxins [7]. The unidentified toxin, which is considered be presented in the head part of the crayfish, may cause injury to the muscle cell membrane and result in severe muscle pain and rhabdomyolysis [6].

In our study, all the 68 patients with crayfish-induced Haff disease were cured and discharged in 8.1 ± 2.3 days after admission and none presented organ failure. Activated charcoal combined with mannitol treatment was showed to be associated with reduced serum enzyme levels, lower anxiety scores, and shorter in-hospital time. These results prove that activated charcoal combined with mannitol has an efficacy for crayfish-caused Haff disease treatment. This may be due to the fact that activated charcoal is an extremely rich pore charcoal structure with multiple pores, which can absorb the toxins in the gastrointestinal tract through physical and chemical adsorption, reduce the concentration of intestinal toxins, and thus promote the toxin excretion [15]. Moreover, its effective absorption capacity can also block the enterohepatic circulation and accelerate the excretion of toxins [16]. Activated charcoal is a common method of gastrointestinal decontamination and typically administered as a single dose. Multiple-dose activated charcoal, with the goal of enhancing toxin elimination rather than reducing absorption per se, is less commonly deployed [17]. In our clinical practice, we chose single-dose treatment of 30 g for these patients. Whether this dosage works best for Haff disease patients needs further exploration. Although generally safe, activated charcoal is not free of risk. The most widely cited risk is pulmonary aspiration [18]. No aspiration was found in these 68 patients probably because all these patients were cooperative. Aspiration risk should be evaluated before activated charcoal administration. Mannitol administered orally has a laxative mechanic effect, favoring fast colonic transit, thus accelerating the evacuation of toxins. Early and adequate fluid infusion is highly recommended for Haff disease treatment. Orally mannitol accelerates intestinal fluid excretion, thus avoiding pulmonary edema or kidney overload which might be caused by aggressive fluid therapy. Besides, oral administration of mannitol can lead to strong contraction of gallbladder sphincter, promote gallbladder emptying, and accelerate the excretion of bile and crayfish toxins [19]. Totally 25 patients presented a rebound rise of CK levels in 36 hours, among which included only 3 from the ACM group (3/22, 14%), while 22 from the non-ACM group (22/46, 48%). The CK rebound rise might be caused by the absorption of residual toxins into the blood.

Many papers reported cases of Haff disease complicated with acute kidney injury, especially when CK > 5000 U/L, so rapid reduction of CK is extremely important for the protection of renal function [20]. The peak levels of CK in most cases of our study were around 10000 U/L, but none of them presented renal injury. This may be related to factors such as short duration of CK peak, quick elimination of toxins, and rapid decrease of CK values. Also, maybe there exists difference between Haff disease caused by crayfish and that induced by other elements.

All patients were presented with local or diffuse myalgia, while seven patients experienced abdominal pain. After 24 hours of treatment, pain scores evaluated by VAS decreased significantly. No statistic difference was found in pain scores between the two groups probably because intravenous remifentanil played a major role in pain relief other than ACM treatment. Patients in the ACM group showed lower anxiety scores compared with the non-ACM group 24 hours after treatments. This may be due to lower CK and CK-MB levels in ACM patients. Doctors and nurses in our department conducted health education about Haff disease to patients on hospital admission. We also told the exact values of these serum enzymes and explained the meaning of these values to patients every morning during hospitalization. Patients in the ACM group showed lower levels of all these serum enzyme values 24 hours after admission as compared with non-ACM patients, which may help them to relieve anxiety.

Since the outbreak of crayfish-induced Haff disease in China in 2010, cases have been consistently reported. We tried to explore the treatment for this disease. However, some limitations apply to this study. First, as it is a retrospective study, some covariates, such as disease severity, other coexisting comorbidities, treatments, and medications, may influence the results. A prospective research study is necessary to validate our findings. Second, it may also restrict the generalization of our results to Haff disease patients that only emergency cases in our hospital were included in this study. High-quality multicenter randomized controlled studies are necessary to be conducted in the future to verify our conclusions because crayfish are consumed daily by so many people in China.

## 5. Conclusion

On the basis of hydration, alkalization of urine, and organ function support treatment, patients with Haff disease caused by crayfish might benefit from activated charcoal combined with mannitol treatment with shorter hospital stay, lower serum CK, CK-MB, AST, ALT, and LDH levels, and lower anxiety scores.

## Figures and Tables

**Table 1:** Demographic and clinical characteristics of Haff disease patients.

Variable	ACM group (*n* = 22)	Non-ACM group (*n* = 46)	*p* value
Age (years)	34.4 ± 9.9	33.6 ± 8.1	0.731

Gender (male/female)	(12/10)	(20/26)	0.392

BMI (kg/m^2^)	21.2 ± 1.5	21.5 ± 1.5	0.389

Time from crayfish eaten to symptom onset (hours)	3.4 ± 1.7	3.9 ± 2.0	0.296

Duration of hospital stays (days)	7.5 (6.0–8.0)	8.0 (6.8–10.0)	0.032

Symptoms			
Myalgia	22	46	
Shortness of breath	6	7	0.237
Nausea or vomiting	5	4	0.11
Abdominal pain	4	3	0.139
Weakness	22	46	
Brown urine/hematuria	3	4	0.531

Medical histories			
Hypertension	3	4	0.531
Coronary heart disease	1	1	0.588
Diabetes mellitus	2	1	0.194

Admission laboratory values			
Creatinine (*μ*mol/L)	68.6 ± 16.1	76.3 ± 20.0	0.119
BUN (mmol/L)	7.3 ± 2.1	7.2 ± 3.1	0.81
WBC (10^9^/L)	8.1 ± 2.8	9.3 ± 2.6	0.091
Hematocrit (%)	43.3 ± 4.9	43.5 ± 6.2	0.878

Remifentanil dose during the first 24 hours of hospitalization (mg)	0.2 ± 0.6	0.4 ± 0.8	0.071

BUN, blood urea nitrogen; WBC, white blood cell; CK, creatine kinase; CK-MB, creatine kinase-muscle/brain; AST, aspartate transaminase; ALT, alanine transaminase; LDH, lactic dehydrogenase.

**Table 2:** Serum CK, CK-MB, AST, ALT, and LDH changes after hospital admission.

	ACM group (*n* = 22)	Non-ACM group (*n* = 46)	*p* value
CK (U/L)			
On admission	5848.2 ± 2710.1	5771.2 ± 2645.5	0.912
12 hours after admission	10354.1 ± 3623.7#	9509.4 ± 4149.1#	0.417
24 hours after admission	3242.3 ± 1218.6#	5443.3 ± 2224.6	0.0001
36 hours after admission	2106.3 ± 1062.5#	5465.0 ± 1536.0	0.0001
48 hours after admission	1082.3 ± 506.3#	3716.5 ± 1129.3#	0.0001
60 hours after admission	483.5 ± 127.4#	2172.1 ± 890.7#	0.0001
72 hours after admission	156.5 ± 47.3#	850.3 ± 263.1#	0.0001

CK-MB (U/L)			
On admission	532.9 ± 125.4	535.5 ± 162.1	0.948
12 hours after admission	353.2 ± 81.5#	445.7 ± 128.7#	0.003
24 hours after admission	306.9 ± 92.8#	365.9 ± 105.2#	0.028
36 hours after admission	207.6 ± 71.9#	295.2 ± 74.1#	0.0001
48 hours after admission	76.2 ± 30.3#	201.5 ± 68.0#	0.0001
60 hours after admission	59.7 ± 18.8#	94.9 ± 29.1#	0.0001
72 hours after admission	49.2 ± 13.2#	58.5 ± 21.8#	0.069

AST (U/L)			
On admission	260.6 ± 67.2	292.8 ± 74.7	0.091
12 hours after admission	175.9 ± 12.0#	217.9 ± 56.7#	0.001
24 hours after admission	115.8 ± 31.5#	179.6 ± 8.2#	0.0001
36 hours after admission	93.3 ± 17.2#	136.4 ± 17.6#	0.0001
48 hours after admission	68.2 ± 13.9#	85.1 ± 15.4#	0.0001
60 hours after admission	57.0 ± 10.1#	77.4 ± 10.9#	0.0001
72 hours after admission	46.8 ± 15.7#	46.8 ± 10.4#	0.981

ALT (U/L)			
On admission	183.1 ± 61.6	184.9 ± 61.3	0.91
12 hours after admission	123.9 ± 15.9#	169.0 ± 50.6	0.0001
24 hours after admission	96.4 ± 16.5#	143.3 ± 41.4#	0.0001
36 hours after admission	85.5 ± 17.7#	128.3 ± 11.1#	0.0001
48 hours after admission	75.1 ± 8.3#	93.3 ± 19.3#	0.0001
60 hours after admission	55.4 ± 7.5#	64.5 ± 9.4#	0.0001
72 hours after admission	39.9 ± 10.0#	50.0 ± 8.6#	0.0001

LDH (U/L)			
On admission	717.7 ± 174.1	739.7 ± 261.4	0.722
12 hours after admission	440.1 ± 141.0#	648.3 ± 221.0	0.0001
24 hours after admission	302.6 ± 102.4#	488.7 ± 152.9#	0.0001
36 hours after admission	215.6 ± 72.0#	429.2 ± 146.7#	0.0001
48 hours after admission	177.9 ± 55.8#	328.8 ± 117.6#	0.0001
60 hours after admission	139.5 ± 47.9#	245.1 ± 68.9#	0.0001
72 hours after admission	94.4 ± 33.8#	164.8 ± 56.1#	0.0001

# represents significant *p* value compared with data on admission. CK, creatine kinase; CK-MB, creatine kinase-muscle/brain; AST, aspartate transaminase; ALT, alanine transaminase; LDH, lactic dehydrogenase.

**Table 3:** Pain and anxiety scores of Haff disease patients.

Variable	ACM group (*n* = 22)	Non-ACM group (*n* = 46)	*p* value
Pain score (VAS)			
At admission	3.2 ± 1	3.2 ± 1.4	0.924
24 hours after admission	1.1 ± 0.9	1.4 ± 1.1	0.348

Anxiety score (SAS)			
At admission	66 ± 7.6	66 ± 6.6	0.981
24 hours after admission	40.7 ± 4.9	44.1 ± 6.3	0.032

VAS, Visual Analogue Scale; SAS, Self-Rating Anxiety Scale.

## Data Availability

Data are available from the corresponding author upon request.
